# Multi-Objective Optimization of Extrusion Parameters for High-Performance Honeycomb Cordierite Ceramics via Orthogonal Design

**DOI:** 10.3390/ma18245550

**Published:** 2025-12-10

**Authors:** Xianpeng Huang, Na Wei, Fengshuang Wang, Xiaoli Zhang

**Affiliations:** 1 Weichai Power Co., Ltd., Weifang 261061, China; wangfengs@weichai.com; 2 College of Materials Science and Engineering, Shandong University of Science and Technology, Qingdao 266590, China; 13561310365@163.com

**Keywords:** cordierite ceramics, extrusion, orthogonal design, mechanical properties, thermal shock resistance

## Abstract

Cordierite diesel particulate filters (DPFs) were prepared using pure cordierite powder with organic binders, sodium silicate aids and pore formers by extrusion technique. The orthogonal test method was adopted to investigate the optimal value of the multi-objective and multi-factor problems. Based on results from statistical analysis, sintering temperature is the most important factor. The optimal parameters for balanced overall performance were determined as a 3 h holding time, 10 wt.% pore former, 12 wt.% sintering aid, and a sintering temperature of 1150 °C, representing a compromise among the individually optimal conditions for porosity, compressive strength, and thermal shock resistance identified by range analysis. The sodium silicate liquid increased and viscosity decreased with the increasing of temperature, which led to the formation of glass phases and the improvement of density. Therefore, with increasing sintering temperature, the porosity and coefficient of thermal expansion decreased. Both the mechanical properties and chemical stability of the prepared samples are strengthened. When the sintering temperature was 1150 °C, the prepared samples with high porosity (56.04%), compressive strength (5.88 MPa), bending strength (13.10 MPa), and low thermal expansion coefficient (CTE, 1.82 × 10^−6^/°C) showed the best comprehensive performance of thermal shock resistance and filtration efficiency. These results demonstrate great potential for DPF applications and provide a reference for the design of other honeycomb ceramics with optimum level of liquid phase.

## 1. Introduction

The exhaust from diesel engines contains a number of air pollutants, including carbon monoxide (CO), hydrocarbons (C_x_H_y_), nitrogen oxides (NO_x_), sulfur oxides (SO_x_) and particulate, which is harmful for human health and environmental safety [1,2]. The diesel particulate filter (DPF) has been developed as an efficient solution for trapping and eliminating soot [3]. The filter operates via a honeycomb ceramic wall that features an interconnected porous network. This structure allows exhaust gases to pass through while trapping soot particles, thereby enhancing filtration performance [4]. As worldwide government regulations associated with particulate matter and NOx emissions continue to become increasingly stringent, the more effective filter to satisfy the particulate controlling requirement is imposed [5]. For a viable DPF, the filter material must simultaneously satisfy several stringent criteria. Industrially, a high and accessible porosity (typically >50–60%) is required to ensure low backpressure and adequate soot storage capacity [6]. The material must also withstand severe thermal stresses during periodic regeneration, where temperatures can rapidly fluctuate by several hundred degrees Celsius [7]. Consequently, a low thermal expansion coefficient (CTE, ideally below 2.0 × 10^−6^/°C) is crucial to minimize thermal stress and prevent cracking, defining the cornerstone of cordierite’s advantage in this application [8]. Cordierite (2MgO·2Al_2_O_3_·5SiO_2_) has been widely used in DPF applications owing to its extremely low thermal expansion coefficient, good thermal stability, and sufficient mechanical strength for monolithic honeycomb structures when combined with appropriate geometric design [9,10]. While its intrinsic strength is lower than that of advanced alternatives like silicon carbide (SiC), its excellent thermo-mechanical stability and cost-effectiveness make it a dominant commercial choice for many filter platforms [11]. However, it is still a great challenge to prepare honeycomb cordierite ceramic with high performance of mechanical and thermal properties.

As we all know, even at stoichiometric cordierite compositions, intermediate phases (impurity crystals and amorphous phase) tend to form at relatively low temperatures [12,13,14], especially when solid-state reaction routes are used for cordierite synthesis. Therefore, the sintering conditions and composition of the cordierite body need to be properly controlled to produce a low-CTE honeycomb. Among the preparation methods, extrusion method is traditionally used to manufacture honeycomb ceramics. The honeycomb ceramics fabricated by extrusion process have high porosity, straight-through channels and readily controllable pore structures [15,16,17]. Particularly, the extrusion method can ensure high production of honeycomb ceramics with low cost. However, during the preparation process, there are high requirements for the viscosity and plasticity of the mud material. Additionally, many influencing factors in this process, such as the content of pore-forming agents, the content of additives, powder particle size, sintering temperature, and holding time can affect the preparation of materials [18,19,20,21]. In general, an exhaustive research method is used to study every factor effect on properties of materials. To efficiently optimize these variables, the orthogonal test method has been adopted. Based on orthogonal design and mathematical analysis, this approach significantly shortens the experimental cycle and facilitates the development of high-performance new materials [22,23].

In this study, honeycomb cordierite ceramics were fabricated by extrusion using a mixture of commercial cordierite powder (to avoid intermediate phase formation), hydroxypropyl methylcellulose, polyvinyl alcohol, sodium silicate as a sintering aid, and carbon powder as a pore-forming agent. This work aims to multi-objectively optimize the extrusion and sintering process for cordierite DPFs, with the goal of achieving high porosity for adequate filtration efficiency and acceptable backpressure, sufficient mechanical strength to withstand assembly and operational stresses, and excellent thermal shock resistance to endure rapid temperature cycles in exhaust environments. An orthogonal experimental design was therefore applied to optimize four key parameters: sintering temperature, holding time, pore-forming agent content, and sintering aid content. The optimal parameter combination was successfully identified. Furthermore, the effect of sintering temperature on pore structure, mechanical properties, thermal shock resistance, and filtration performance was systematically investigated.

## 2. Materials and Methods

### 2.1. Raw Material

Cordierite (2MgO·2Al_2_O_3_·5SiO_2_) powders with a particle size of d_50_ = 45 μm (Jiangxi Henghao New Material Technology Co., Ltd., Jiangxi, China) were used as the main ceramic phase. The purity was over 97% according to the supplier’s information. Carbon powder (C, Fuchen Tianjin Chemical Reagent Co., Ltd., Tianjin, China) served as the pore-forming agent. Hydroxypropyl methylcellulose (HPMC, Tianjin Huasheng Chemical Reagent, Tianjin, China) was added as a plasticizer to improve extrusion rheology; glycerol (C_3_H_8_O_3_, Tianjin Fuyu Fine Chemicals, Tianjin, China) acted as a lubricant to reduce friction during shaping. Sodium silicate (Na_2_SiO_3_, Aladdin Reagent Co., Ltd., Shanghai, China) was employed as a sintering aid to promote liquid-phase densification, and polyvinyl alcohol (PVA, Aladdin Reagent Co., Ltd., Shanghai, China) was used as a binder to enhance green strength.

### 2.2. Preparation of Honeycomb Shaped Cordierite

**Figure 1:**
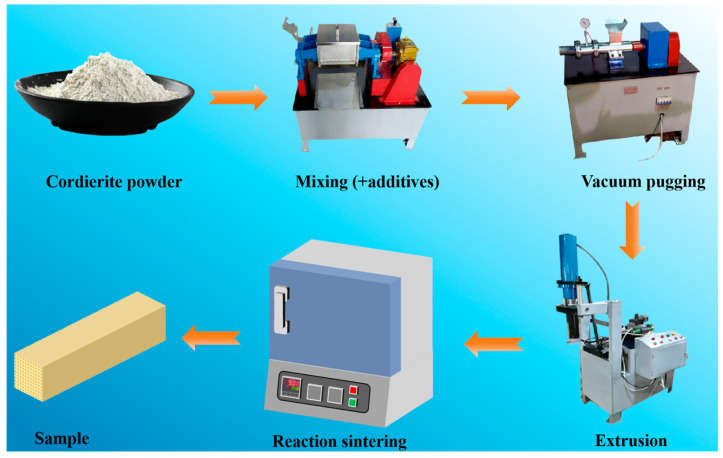
Schematic of extrusion molding method for fabrication of honeycomb shaped cordierite ceramics.

Cordierite powder was mixed with carbon powder (0–15 wt%) for 0.5 h in a laboratory ceramic mixer (model HN-5, Zhongbo Ceramic Machinery Factory, Henan, China), followed by addition of sodium silicate (6–15 wt.%). A binder of 5 wt.% aqueous PVA solution (based on total powder mass) was introduced, along with 8 wt.% HPMC, 32 wt.% deionized water, and 5 wt.% glycerol (relative to cordierite mass). For instance, in one representative formulation in the orthogonal test design, 860 g of cordierite powder was mixed with 50 g of carbon powder (5 wt.%) and 90 g of sodium silicate (9 wt.%). A binder of 5 wt.% aqueous PVA solution (equivalent to 50 g based on the total powder mass) was introduced. Additionally, 68.8 g of HPMC (8 wt.% relative to cordierite mass), 275.2 g of deionized water (32 wt.% relative to cordierite mass), and 43 g of glycerol (5 wt.% relative to cordierite mass) were added. The mixture was kneaded in the same mixer for 0.5 h to form homogeneous clay, then processed in a vacuum pug mill (model S-48, Zhongbo Ceramic Machinery Factory, China) to remove air bubbles and impurities. After aging in a sealed container for 48 h, the clay was shaped by extrusion using a laboratory ceramic tube-extrusion machine (model LWJ-63, Zhongbo Ceramic Machinery Factory, China). The dried samples were sintered at 1000–1300 °C for 1–4 h to obtain honeycomb cordierite ceramics. The preparation process was shown in Figure 1.

### 2.3. Orthogonal Experimental Design (OED)

The OED is an effective mathematical method for multi-factor experimental design, which can fully explore the interaction relationships between different factors and their levels within a limited number of trials. Its characteristics include uniform and independent level settings for each experimental factor, ensuring that every level of each factor is thoroughly examined across all trials, thereby avoiding errors caused by factor cross-interference. In the research, four factors, including sintering temperature (A), holding time (B), pore-forming agent content (C), and sintering aid content (D) were selected as the orthogonal design factors and each factor was selected in four levels. The parameter ranges for the orthogonal design of sintering temperature (800–1150 °C), pore-former content (0–15 wt.%), and sintering aid content (6–15 wt.%) were selected based on general ceramic-processing principles to explore a practical sintering window while accounting for the distinct roles of each component in tailoring porosity and densification. Factors and levels for orthogonal test design were shown in Table 1.

### 2.4. Characterization

The phase compositions of the as-prepared samples were analyzed by X-ray diffraction (XRD). The morphology of fractured surface was obtained by scanning electron microscopy (SEM, NANO450, FEI, Hillsboro, OR, USA). Open porosity was measured by the Archimedes method. A mercury intrusion porosimeter (Micromeritics AutoPore V 9600, Norcross, GA, USA) was used to analyze the pore size distribution and porosity of samples sintered at different temperature. Compressive strength was evaluated on 20 mm × 20 mm × 20 mm samples with an MTS E45.305 universal testing machine (Eden Prairie, MN, USA). Bending strength was measured by the three-point bending method on an INSTRON-5582 testing machine (Norwood, MA, USA). The CTE was evaluated from room temperature to 800 °C using a thermal dilatometer (ZRPY-1400, Xiangtan Xiangyi Instrument Co., Ltd., Xiangtan, China). The average CTE were calculated from the intercepted linear portion (325–800 °C).

The thermal shock resistance was assessed based on the methods stipulated in standards JC/T 2396-2017 [24] and GB/T 25994-2010 [25]. Samples with size of 40 mm × 40 mm × 100 mm were subjected to thermal cycling in a muffle furnace at 650 °C for 30 min, followed by rapid removal and natural cooling to room temperature. Cycling was continued until crack initiation was observed on the honeycomb walls. Chemical stability was assessed by measuring the mass loss after corrosion in acidic and alkaline solution. Prior to testing, all samples were ultrasonically cleaned and oven-dried to a constant mass (M_1_). The samples were then subjected to immersion in 20 vol.% H_2_SO_4_ and 1 vol.% NaOH solutions at 80 °C for 1 h, respectively. Subsequently, the samples were dried and weighed (M_2_). The mass loss rate (R) was calculated according to the equation of R = (M_1_ − M_2_)/M_1_ × 100%. A gravimetric method was employed to determine the filtration efficiency of cordierite samples against carbon aerosol. Briefly, 0.05 g of carbon powder was dispersed in anhydrous ethanol to form a 2000 ppm stock solution. Prior to testing, each cordierite sample was dried at 110 °C for 10 h and its initial mass (M_0_) was recorded. The sample was then installed in the home-made test apparatus, and the carbon black aerosol was generated by injecting the solution into an atomizer, with a nitrogen carrier gas flow set at 100 mL/min. After the test, the sample was dried thoroughly to remove the ethanol and weighed again (M_1_). The mass gain (∆M_1_ = M_1_ − M_0_) represents the mass of captured carbon black. Additionally, the carbon aerosol was dried thoroughly to remove the ethanol and weighed again (M_3_). The mass loss of the carbon powder was ΔM_2_ (∆M_2_ = 0.05 g − M_3_). The filtration efficiency (η) was calculated using the following formula of *η* = (ΔM_1_/ΔM_2_) × 100%.

## 3. Results

### 3.1. Orthogonal Test Results

According to the predetermined factors and levels, the orthogonal table was designed using the SPSSAU data science analysis platform (source: https://spssau.com/, accessed on 10 March 2024) [26]. An L_16_(4^4^) Taguchi orthogonal array (denoting 16 experimental trials with four factors each at four levels) was adopted to investigate the effects of these factors on the pore structure and properties of cordierite ceramics. To evaluate the influence of different factor levels, porosity, compressive strength, and thermal shock resistance were measured, as summarized in Table 2. The effects of various factors on these properties were assessed and the optimal combination of factor levels was determined using range analysis. Table 3 provides the range analysis of this orthogonal experiment, in which *K_i_* (level i = 1, 2, 3, 4) and *R* are important parameters. *K_i_* is defined as the average value of the test performance at the four levels of the corresponding factor, and R is defined as the difference between the *K_max_* and *K_min_* values in the corresponding factor column. For example, for porosity, when the factor A is 1 (800 °C), *K*_1_ = (76.46 + 72.98 + 61.2 +58.94)/4, *R* = max{67.40, 65.23, 66.26, 59.72} − min{67.40, 65.23, 66.26, 59.72} = 7.68. By comparing the K values, the optimal level for each factor can be identified. The R value reflects the fluctuation range of the *K* values; thus, a larger *R* value indicates a more significant influence of that factor on the experimental results [27,28].

In Table 3, it can be found that the greatest influence on porosity was holding time, followed by sintering temperature, sintering aid content, and pore-forming agent content, namely B > A > D > C. By observing the maximum values of K_1_, K_2_, K_3_ and K_4_, the optimal combination of process parameters were A1 (800 °C), B_1_ (1 h), C_4_ (15 wt.%) and D_1_ (6wt.%). The sintering temperature was the biggest influence on the compressive strength, followed by pore-forming agent content, holding time, and sintering aid content, that is A > B > C > D. It should be noted that for compressive strength, the optimal parameters were A_4_ (1150 °C), B_2_ (2 h), C_1_ (0 wt.%) and D_4_ (15 wt.%). The four factors affecting thermal shock resistance were ranked in descending order of influence: sintering temperature, pore-forming agent content, holding time, and sintering aid content, that is A > C > B = D. To sum up, the main factor that affects the compressive strength and thermal shock resistance is the sintering temperature. For thermal shocks, the optimal parameters were A_4_ (1150 °C), B_3_ (3 h), C_2_ (5 wt.%) and D_3_ (12 wt.%).

**Figure 2:**
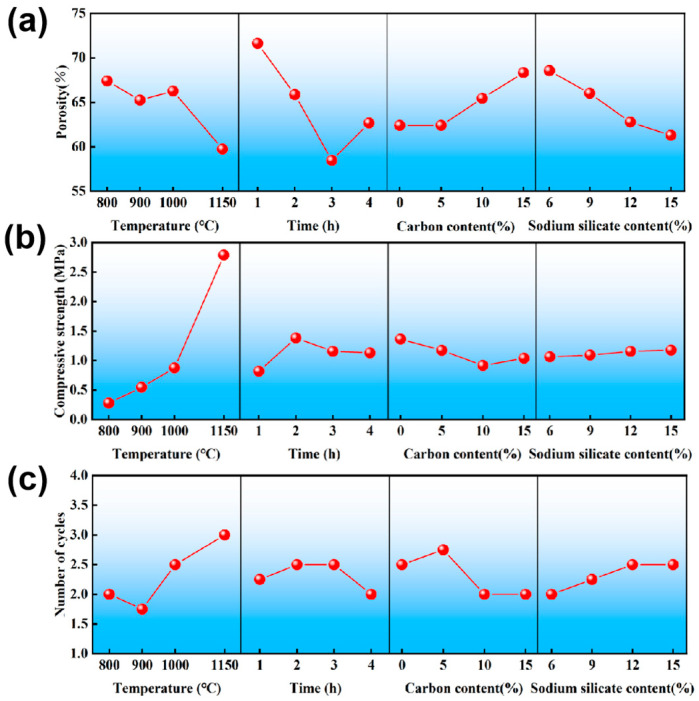
Influence of four factors on the (**a**) porosity, (**b**) compressive strength and (**c**) thermal shock resistance.

Figure 2 is drawn according to the K values of the porosity, compressive strength, and thermal shock resistance in Table 3. As shown in Figure 2a, the porosity of the samples decreased gradually with the increase in sintering temperature and the extension of holding time, which can be attributed to the enhanced density of the samples. Moreover, a higher content of sodium silicate as the sintering aid promoted the formation of a liquid phase, which not only filled the pores but also facilitated particle rearrangement and densification. Consequently, the overall porosity decreased significantly. In contrast, porosity exhibited an increasing trend as the content of the pore-forming agent rises. Figure 2b reveals a clear negative correlation between compressive strength and porosity. As the sintering temperature increased, porosity showed a decreasing trend while compressive strength progressively rises. This relationship was typified at 1150 °C, where the porosity decreased to 59.72% and the compressive strength reached its maximum of 2.70 MPa. Meanwhile, as illustrated in Figure 2c, the thermal shock resistance improved notably with increasing sintering temperature. The maximum thermal shock resistance was achieved at a sintering temperature of 1150 °C. However, beyond this point, cracks began to appear after three thermal cycles.

Taking into account the influencing factors and parameters of various properties, it can be seen from the factors affecting thermal shock resistance and compressive strength that sintering temperature is the main influencing factor, and the effect of sintering temperature on microstructure and properties was investigated in the following research. The holding time was set to 3 h, as it represents the optimal level for thermal shock resistance. Analysis of the optimal parameters for compressive strength and thermal shock resistance reveals that when the pore-forming agent content increases from 0 wt.% to 5 wt.%, the thermal shock resistance improves. This indicates that introducing a small number of pores can significantly enhance thermal shock resistance without a notable increase in overall porosity. Accordingly, a pore-forming agent content of 10 wt.% was selected to balance porosity requirements, even though the thermal shock resistance may slightly decrease at this level. In addition, a sintering aid content of 12 wt.% was identified as the optimal value for thermal shock resistance. Based on these considerations, the final optimized combination of process parameters was determined as follows: holding time of 3 h, pore-forming agent content of 10 wt.%, and sintering aid content of 12 wt.%.

### 3.2. Effects of Sintering Temperature

**Figure 3:**
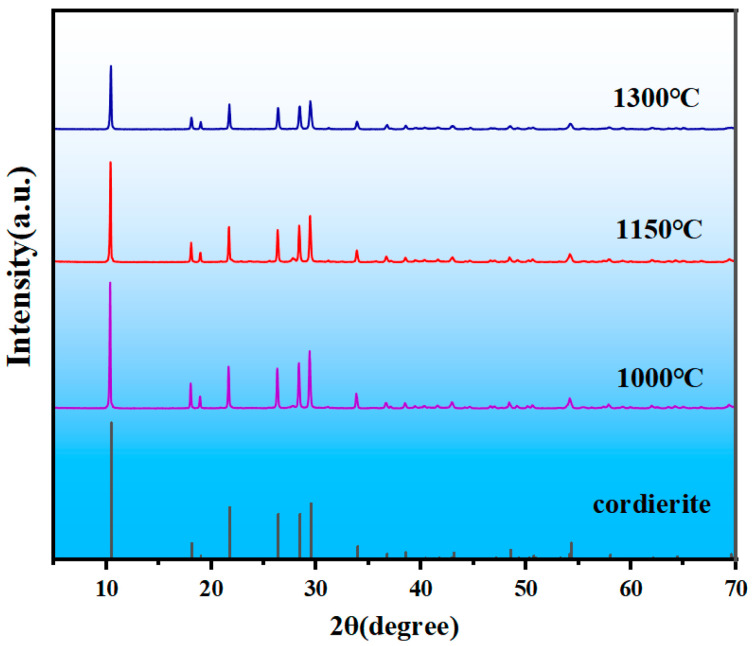
XRD patterns of samples sintered at 1000 °C, 1150 °C and 1300 °C.

Figure 3 presents the XRD patterns of samples sintered at 1000 °C, 1150 °C, and 1300 °C, in which all diffraction peaks correspond to crystalline cordierite (hexagonal α-type structure, space group of P6/mcc, JCPDS#82-1887). With increasing sintering temperature, a progressive weakening of the cordierite diffraction peaks is observed. This trend is attributed to the growth of an amorphous silicate glass phase derived from the sodium silicate sintering aid, which forms a viscous liquid at elevated temperatures. At 1000 °C, the sodium silicate liquid exhibits limited reactivity and fluidity, preserving most crystalline cordierite grains and yielding the highest peak intensity [29]. As the temperature rises to 1150 °C, the increased fluidity of the liquid phase promotes cordierite dissolution and the formation of a thicker glassy intergranular layer, thereby raising the amorphous fraction and further reducing diffraction intensity. At 1300 °C, the highly mobile liquid induces extensive dissolution, penetrating into grain interiors and maximizing the glassy phase volume, which leads to the lowest observed cordierite peak intensity. It should be noted that while pure cordierite systems typically exhibit enhanced crystallinity above 1200 °C, the presence of the silicate additive in this study alters the phase evolution path, favoring amorphous phase formation and thereby reducing the coherent scattering volume of the crystalline cordierite.

**Figure 4:**
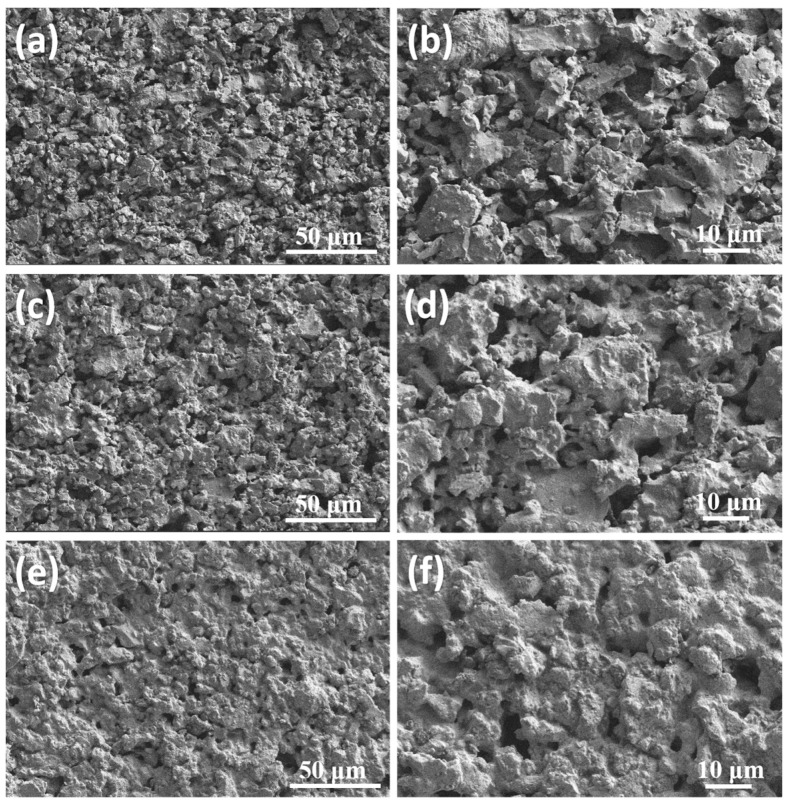
SEM images of samples sintered at different temperatures. (**a**,**b**) 1000 °C, (**c**,**d**) 1150 °C and (**e**,**f**) 1300 °C.

Figure 4 shows the SEM images of samples sintered at different temperatures. The prepared cordierite ceramics presented a three-dimensional interconnected pore structure. As shown in Figure 4a,b, when the temperature was 1000 °C, the particles were mainly in point contact, and the sintered sample showed a loose structure. With the sintering temperature increased, the sintered neck became more and more obvious (Figure 4c,d). However, when the temperature reached 1300 °C, the surface of the particles was bonded by a large amount of liquid phase, the pores were significantly reduced, and the density was increased.

The pore structure develops in three key stages. Initially, between room temperature and 600 °C, organics and carbonaceous substances decomposed, releasing gaseous products and creating an interconnected open-pore network, which established the high porosity of the green body. Subsequently, between 600 °C and 1150 °C, molten sodium silicate formed a wetting liquid phase. A reaction at the cordierite–liquid interface produced a glassy phase that bonds adjacent particles [30]. Finally, during the high-temperature holding stage, the sintering mechanism became strongly temperature-dependent. At 1000 °C, viscous flow induced particle rearrangement, resulting in a weak, highly porous structure. As the temperature rises to 1150 °C, a less viscous liquid facilitated a dissolution–precipitation process, promoting strong interparticle connections and forming a robust porous network. In contrast, at 1300 °C, solid-state sintering combined with liquid volatilization led to over-sintering, resulting in pore closure and a sharp decrease in porosity.

**Figure 5:**
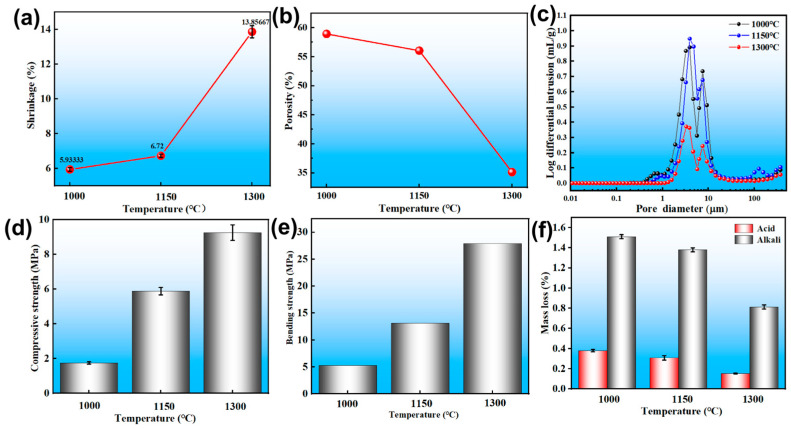
The pore structure and properties of the samples sintered at different temperatures. (**a**) Shrinkage, (**b**) porosity, (**c**) pore size distribution, (**d**) compressive strength, (**e**) bending strength and (**f**) corrosion resistance.

The shrinkage rate exhibited a linear increase trend with the rise in sintering temperature, which was negative to the trend of the open porosity (Figure 5a). This correlation stems from the nature of the sintering process, which inherently involves densification and volume reduction. Prior to sintering, the gaps between particles in the honeycomb ceramic support were predominantly open pores. As the sintering temperature increased, a liquid phase formed, progressively filling these open pores and leading to pore closure, thereby inducing shrinkage of the ceramic structure. With further temperature increase, the volume of the liquid phase expanded, intensifying the shrinkage. As shown in Figure 5b, the open porosity of the cordierite ceramics, as determined by mercury intrusion porosimetry, exhibited a temperature-dependent evolution. When the sintering temperature was raised from 1000 °C to 1150 °C, the porosity decreased moderately from 58.93% to 56.04%. This gradual reduction can be attributed to the increasing volume of the liquid phase, which promotes the initial closure of fine pores and pore throats within the interconnected network [31]. However, when the temperature reached 1300 °C, the porosity dropped markedly to 35.13%. This accelerated decline is consistent with the sharply decreased viscosity of the liquid phase at higher temperatures, which enhances capillary-driven rearrangement and leads to the rapid elimination of small pores, as further corroborated by the pore size distribution results (Figure 5c). Correspondingly, the average pore size increased from 3.42 μm to 4.67 μm over the temperature range from 1000 °C to 1300 °C, indicating a shift toward a more open, coarser pore structure. These results and the observed pore coarsening are in accordance with the microstructural evolution revealed by SEM analysis (Figure 4).

Figure 5d,e shows the compressive strength and flexural strength of the prepared samples, respectively. Both properties increased almost linearly with rising sintering temperature. Specifically, the compressive strengths of samples sintered at 1000 °C, 1150 °C, and 1300 °C were 1.75 MPa, 5.88 MPa, and 9.25 MPa, respectively, while the corresponding bending strengths were 5.27 MPa, 13.10 MPa, and 27.90 MPa. This enhancement in mechanical strength is attributed to the reduction in pores within the ceramic body, which is consistent with the earlier observed decrease in open porosity.

As shown in Figure 5f, the results indicated that the corrosion rate decreased with increasing sintering temperature, which was primarily attributed to a reduction in porosity of the as-prepared samples. Furthermore, the mass loss was consistently more severe in the alkaline solution than that in the acidic solution. This difference stems from the aggressive reaction of OH^−^ ions with the SiO_2_ network in the silicate structure, which generates soluble silicates and leads to widespread dissolution [32]. In contrast, the cordierite structure remained relatively stable in H_2_SO_4_, owing to its lower susceptibility to attack by H^+^ ions [33,34].

**Figure 6:**
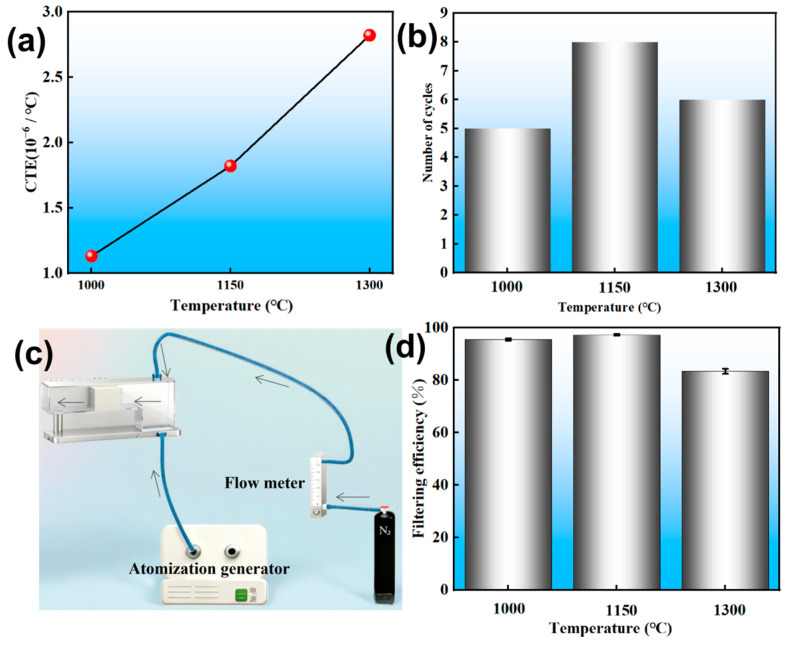
The CTE (**a**), thermal shock resistance (**b**), schematic diagram of filtration device (**c**) and filtering efficiency (**d**) of the samples sintered at different temperatures.

As shown in Figure 6a, the CTE increased from 1.13 × 10^−6^/°C to 2.82 × 10^−6^/°C with increasing sintering temperatures. The CTE of cordierite ceramics stemmed mainly from the glassy and cordierite phases. With the increasing of sintering temperature, the high fraction of glassy phases resulted in the relatively higher CTE values. Furthermore, the presence of an amorphous phase not only increased the CTE but could also induce thermal stress cracking when DPFs were exposed to the rapid thermal cycles of vehicle exhaust systems [35]. In Figure 6b, the honeycomb ceramic sintered at 1150 °C exhibited optimal thermal shock resistance, sustaining eight cycles before crack initiation, whereas the sample sintered at 1000 °C failed after only five cycles. The inferior performance at 1000 °C was due to weak interparticle bonding, which offered low resistance to thermal stress. Conversely, the sample sintering at 1150 °C promoted the formation of a reinforcing glass phase that created strong necks between particles, thereby enhancing fracture strength. The decline in performance at 1300 °C was ascribed to an increased thermal expansion coefficient [36]. Figure 6c shows the schematic diagram of filtration device. As illustrated in Figure 6d, the filtration efficiency first increased and then decreased with increasing sintering temperature, with a maximum value of 97.27% observed at 1150 °C. This decline was attributed to the concomitant reduction in porosity and specific surface area, coupled with an increase in average pore diameter.

## 4. Conclusions

In conclusion, honeycomb cordierite ceramics were fabricated by extrusion and optimized using an L16 (4^4^) orthogonal experiment. Sintering temperature was identified as the most significant factor, with the optimal parameters determined as 3 h holding time, 10 wt.% pore-forming agent, and 12 wt.% sintering aid. While range analysis indicated that maximum compressive strength favored 0 wt.% pore former and 15 wt.% sintering aid, the selected combination (10 wt.% and 12 wt.%, respectively) was chosen to simultaneously retain high porosity and superior thermal shock resistance, thereby achieving an optimal balance of the three competing key properties for DPF application. Further investigation into sintering temperature revealed that as temperature increased from 1000 °C to 1300 °C, porosity decreased sharply from 58.93% to 35.13%, which was attributed to enhanced liquid phase formation and pore coalescence. This densification led to improved mechanical properties. In addition, the mechanical properties and chemical stability of the prepared samples are strengthened with increasing sintering temperature. The sample sintered at 1150 °C with low coefficient of thermal expansion (1.82 × 10^−6^/°C) achieved an optimal balance, exhibiting excellent thermal shock resistance and high filtration performance.

## Figures and Tables

**Table 1:** Factor and level table.

Level	Factor Level			
	Sintering Temperature (℃)	Holding Time (h)	Pore-Forming Agent Content (wt.%)	Sintering Aid Content (wt.%)
	A	B	C	D
1	800	1	0	6
2	900	2	5	9
3	1000	3	10	12
4	1150	4	15	15

**Table 2:** The design and results of orthogonal table of L_16_(4^4^).

No.	Factors				Porosity (%)	Compressive strength (MPa)	Thermal Shock Cycles
	A	B	C	D			
1	800	1	0	6	76.46	0.20	2
2	900	1	5	9	69.45	0.25	2
3	1000	1	10	12	71.29	0.45	2
4	1150	1	15	15	69.27	2.38	3
5	800	2	10	9	72.98	0.23	2
6	900	2	15	6	73.22	0.72	1
7	1000	2	0	15	60.01	1.42	3
8	1150	2	5	12	57.24	3.17	4
9	800	3	15	12	61.20	0.26	2
10	900	3	10	15	56.91	0.46	2
11	1000	3	5	6	63.92	0.82	3
12	1150	3	0	9	51.79	3.09	3
13	800	4	5	15	58.94	0.45	2
14	900	4	0	12	61.34	0.75	2
15	1000	4	15	9	69.83	0.81	2
16	1150	4	10	6	60.58	2.52	2

**Table 3:** Range analysis of properties of cordierite ceramics obtained based on orthogonal test.

	Parameter	A	B	C	D
Porosity (%)	K_1_	67.40	71.62	62.40	68.55
	K_2_	65.23	65.86	62.39	66.01
	K_3_	66.26	58.46	65.44	62.77
	K_4_	59.72	62.67	68.38	61.28
	R	7.68	13.16	5.99	7.27
Compressive strength (MPa)	K_1_	0.28	0.82	1.36	1.06
	K_2_	0.54	1.38	1.17	1.09
	K_3_	0.88	1.16	0.92	1.16
	K_4_	2.70	1.13	1.04	1.18
	R	2.42	0.56	0.44	0.12
Thermal shock cycles	K_1_	2	2.25	2.5	2
	K_2_	1.75	2.5	2.75	2.25
	K_3_	2.5	2.5	2	2.5
	K_4_	3	2	2	2.5
	R	1.25	0.5	0.75	0.5

## Data Availability

The original contributions presented in this study are included in the article. Further inquiries can be directed to the corresponding authors.
